# Socioeconomic Status and Stress Rate during Pregnancy in Iran

**DOI:** 10.5539/gjhs.v6n4p254

**Published:** 2014-04-22

**Authors:** Sara Shishehgar, Mahrokh Dolatian, Hamid Alavi Majd, Maryam Bakhtiary

**Affiliations:** 1 Centre for Cardiovascular & Chronic Care, Faculty of Health, UTS, Australia; 2 Nursing and Midwifery Department, Shahid Beheshti University of Medical Sciences, Tehran, Iran; 3 Paramedics Department, Shahid Beheshti University of Medical Sciences, Tehran, Iran; 4 Department of Psychiatric, Taleghani Hospital, Shahid Beheshti University of Medical Sciences, Tehran, Iran

**Keywords:** socioeconomic status, pregnancy, stress, Iran

## Abstract

**Background:**

Stress during pregnancy can have serious adverse outcomes on the mother, the fetus, newborn, children and even adolescents. Socioeconomic status has been recognized as a predictor of stress amongst pregnant women.

**Objectives:**

The first aim of this study was to investigate the role of socioeconomic status in pregnancy stress rates. The second aim was to examine the most important items of socioeconomic status including monthly family income, husband occupational status as well as mother’s educational level and their influence on the rate of maternal stress.

**Methods:**

This study was cross-sectional research and was conducted on 210 pregnant women in three trimesters of pregnancy who attended Shahryar hospital for prenatal care between August-October 2012. They completed two questionnaires of Socioeconomic Status and Specific Pregnancy Stress. Collected data were analyzed by SPSS version 19 including T-test, one-way ANOVA and Spearman correlation.

**Results:**

In this study, we considered family income, education and husbands’ occupations as the most important variables which may influence perceived stress during pregnancy. The mean age of women was 27±4.8 years. The final result showed that there is no significant relationship between SES and pregnancy stress level (P>0.05), while we found a significant relationship, as well as indirect correlation between husbands’ occupational status and pregnancy stress (P<0.05, r= -0.364).

**Conclusion:**

Further investigations may be considered for extending the results to all pregnant women. Thus, health officials and universities should finance other studies to investigate this fact and whether other dimensions of SES influence pregnancy stress levels or not.

## 1. Introduction

Pregnancy is recognized as a stressful event in a woman’s life that needs huge psychological adjustment (Elsenbruch et al., 2007), however, it is often an exciting time (Talley, 2013). Pregnancy stress is defined as “the imbalance that a pregnant woman feels when she cannot cope with demands, which is expressed both behaviorally and physiologically” (Ruiz & Fullerton, 1999). Also, Littletone et al. (2007) have determined “pregnancy-specific anxiety” as concerns, worries about pregnancy, fear of childbirth, infant well-being and future parenting. It is well documented that stress during pregnancy can have a huge number of maternal as well as neonatal adverse effects. Many researchers have asserted that maternal stress during pregnancy has been associated with preterm labor, low birth weight, Preeclampsia, abortion, suppressing the Immune system, nausea as well as vomiting, increase in episiotomy and newborn infection and adverse physical and mental outcomes (Bale et al., 2010, Bilbo & Schwarz, 2009; Divney et al., 2012; Ruiz & Fullerton, 1999). Also, hyperactivity for children of school age and behavioral issues could be the results of maternal stress. Additionally, maternal stress has been defined as a potential predictor of depression during and after pregnancy (Zelkowitz et al., 2004; Holzman et al., 2006; Lancaster et al., 2010; Records & Rice, 2007) and schizophrenia among male offspring (van Os & Selten, 1998; Khashan et al., 2008; Bale et al., 2010). Apart from the problems mentioned, Public health could suffer from the short and long-term effects of maternal stress (Kingston et al., 2012). According to the literature, prevalence of pregnancy stress is reported 33-37% in England and 5-7% and Sweden. While Salari et al. (2005) estimated the prevalence of severe and mild maternal stress 16.7 and 13.6 percent respectively with Iranian women. Many factors have been identified as effectual elements increasing or decreasing the stress level. These include social support, quality of life and socioeconomic status (Lancaster et al., 2010; Lau & Yin, 2011; Dunkel Schetter, 2011; Divney et al., 2012; Da Costa et al., 2010). Among these three mentioned factors, socioeconomic status plays a crucial role in the stress perceived by pregnant women (Kingston et al., 2012, Whitehead et al., 2003). This means that pregnant women with low socioeconomic status can experience greater stress during pregnancy rather than those with higher socioeconomic status (Kingston et al., 2012, Lever, 2008). However, it is documented in some studies that gender, marital and socioeconomic status influence the stress perceived by people. Whereas, others believe that there is no straight relationship between SES and perceived stress during pregnancy (Matthews et al., 2010). Regarding the importance of the relationship between maternal stress and its adverse outcomes, there is an existing gap in the literature about decreasing or increasing impacts of SES on prenatal stress. Therefore, we conducted the present study to explore the relationship of socioeconomic status and the rate stress during pregnancy.

## 2. Method and Materials

This cross-sectional study was conducted in August-October 2012. The study population were all pregnant women, according to convenience sampling (that met the study inclusion criteria), attending the Shahryar hospital affiliated to Social Security Organization located in the west of Tehran, Iran. A purposeful sampling methodology was selected in order to recruit required samples. The aim of purposeful sampling was to select pregnant women in various trimesters of pregnancy to gain miscellaneous experiences of stress. The required sample size, using correlation formula, was 210 patients that were selected by purposeful convenient sampling. Participants were all pregnant women who also included the following criteria: women in the first or second pregnancy, with a single fetus, without any medical, mental, or disabled spouse or child, and no major life event in their last six months, non-smoker or drug user and those who had performed the necessary pregnancy care. After getting permission from Shahid Beheshti Chancellor and Social Security Organization, sampling was started. Also, before commencement, women were informed about the purpose of study and were asked to sign the consent form if were willing to take a part. They were assured that despite entering in the study they could withdraw any time they wished and their information would be kept confidential. Finally, we tried to consider the privacy of individuals.

### 2.1 Data Collected Through Distinct Pregnancy Stress and Socioeconomic Status Questionnaires

#### 2.1.1 Pregnancy Stress Questionnaire

Pregnancy stress scale consisted of 51 questions in six domains including health, personal and family, environmental, financial, religion, and how others think about pregnant mother. This question¬naire has designed with 5-point Likert style of zero (minimum) to 204 (maximum). In all areas grading is done as follows 0 = 0, low = 1, medium = 2, high = 3 and very high = 4. After the data collection and conversion, num¬bers categorized into three grades: mild stress = 0-33.3%, average stress = 33.4-66.3%, severe stress = 66.4-100%.

The Specific stress in pregnancy ques¬tionnaire has been validated by Salari et al. with test-retest and reliability of that has been determined with Cronbach alpha coefficient = 0.75 (Salari et al., 2005).

#### 2.1.2 Socioeconomic Status Questionnaire

Socio-economic status was assessed by a questionnaire including current marital status, pregnant woman’s and spouse’s occupation and education level, monthly income, their place of residence and number of people per household, cost per square meter of their house, facilities and leisure (having a private car and computer). Participants divided in three groups of low (<4000000R), medium (4000000-8000000R) and high (>8000000R) income. Mothers were set up into two groups, low and well-educated. Low-educated respondents were those with education under a diploma level, however, mothers with an academic education were considered as well-educated participants.

In addition, the partners’ jobs were defined as low, middle and high-class jobs. Laborers and less-educated workers were categorized as low-class jobs. However, bazaar merchants, managers of private, owners of transport companies were identified as middle-class occupations. In addition, participants who their spouses had jobs due to a high level education such as physicians, engineers, teachers and large-scale factory owners were placed in high-class jobs category. Using factor analysis and summary index, the correlation of these parameters was determined with the total score of 0.87. The total standardized score for all subjects was calculated, using the Kappa test, its compliance with normal summary index was investigated (Garmaroudi & Moradi, 2010). The potential maximum score in the summarized index was 46 marks. After the data collection and conversion, the total socioeconomic status was categorized in two groups with score under 16 versus 16 and upper. These two groups were named inappropriate versus appropriate, respectively.

### 2.2 Data Analysis

The collected data were analyzed by SPSS.19 using one-way ANOVA for exploring the relationship between socioeconomic status as a whole and its items with pregnancy stress rate and its six domains. In addition, we used T-test for analyzing the relationship between the stress level and mother’s education during pregnancy. Spearman test was applied to show the exact correlation between variables, which were significant. Pvalue of < 0.05 was considered as significant level statistically.

## 3. Results

This study was conducted on 210 pregnant women with the mean age 27±4.8 years with equal numbers (N=70) in three trimesters of pregnancy. The majority (90.5%) were city dwellers, educated at high school diploma level (51.4%), housewives (89%), Az¬ari (40.5%), and had wanted/planned pregnancies (78.6%) (see details in Table 1).

**Table 1:** General information of pregnant women (n=210) attending to Shahryar hospital including SES

Variables	No (%) (N=210)	Mean±SD
**Age, Years**		27±4.8
<25	87(41.4)	
25-30	73(34.8)	
30-35	40(19)	
>35	10(4.8)	
**Education**		
Low-educated	155(73.8)	
Well-educated	55(26.2)	
**Husband Occupation**		
Low class job	26(12.4)	
Medium class job	144(68.6)	
High class job	40(19)	
**Monthly Income**		
<4000000R	49(23.3)	
4000000-8000000R	114(54.3)	
>8000000R	47(22.4)	
**Residential Area**		
City	190(90.5)	
Rural	20(9.5)	
**Wanted pregnancy**		
Wanted	165(78.6)	
Unwanted	45(21.4)	
**Ethnicity**		
Azari	85(40.5)	
Fars	69(32.9)	
Kord	22(10.5)	
Lor	18(8.5)	
Gilak	16(7.6)	
***Total***	***210***	

According to our results, there was no significant relationship between socioeconomic status and total perceived stress as well as its six main dimensions (P >0.05) (see Table 2).

**Table 2:** The relationship of SES as a whole and all six dimensions of pregnancy stress

Variable	Health (Pvalue)	Religion (Pvalue)	Financial (Pvalue)	Environmental (Pvalue)	Personal-Family (Pvalue)	How others think (P value)
Socioeconomic Status
	0.415					
	_	0.226				
	_	_	0.281	0.198		
	_	_	_			
	_	_	_	—	0.695	
	_	_	_	_	—	0.546

In this study, we considered family income, husband occupational status and education as the most important variables might influence perceived stress during pregnancy. Table 3 indicates our findings in relation to these three factors.

**Table 3:** Relationship and correlation of various stress levels and the most important variables of SES

Variables	Mild stress (N=68)	Moderate stress (N=116)	Severe stress (N=26)	ANOVA & T-test	Correlation
**Income**					
Low-income	19(38.8%)	24(49%)	6(12.2%)	Pvalue>0.05	r= 0.040
Moderate-income	37(32.45%)	60(52.6%)	17(14.95%)		
High-income	12(25.5%)	32(68.1%)	3(6.4%)		
**Education**					
Low-educated	53(34.2%)	81(52.25%)	21(13.55%)	Pvalue>0.05	r= 0.017
High-educated	15(27.3%)	35(63.6%)	5(9.1%)		
**Husband Occupation**				Pvalue<0.05	r= -0.364*
Low class job	3(11.55%)	7(26.9%)	16(61.55%)		
Medium class	47(32.65%)	88(61.1%)	9(6.25%)		
High class	18(45%)	21(52.5%)	1(2.5%)		
***Total***	*68*	*116*	*26*		

* Correlation is significant at the 0.05 level

As it is obvious in Table 3, there is a significant relationship between the type of husbands’ occupation and perceived stress by mothers. Similarly, an indirect correlation exists between these two variables (see details in Table 3).

## 4. Discussion

The Socioeconomic status has been considered as following items: woman and her spouse’s education, woman and her spouse’s occupation, income, their place of residence and number of people per household, value of per square meter of their house, facilities and leisure (having a private car and computer) (Garmaroudi & Moradi, 2010). In this study, all efforts have done to show if mothers’ socioeconomic status has a strong influence on pregnancy, delivery and newborn outcomes with effecting on the maternal stress rate. Since the most significant dimensions of SES consist of family Income, husband occupation and mother’s education, we tried to consider these three variables in the separate sections (Gallo et al., 2012; Morrison, Najman, Williams, Keeping, & Andersen, 1989). Contrary to Lever’s study (2008) demonstrating a direct relationship between SES and individual’s stress, nothing significant was found between family income and mother’s education with perceived pregnancy stress rate (Lever, 2008).

In term of monthly family income, Lever (2008), Goyal (2010) and Gallo (2012) illustrated inconsistent outcomes. They found that family income influences individual’s stress rate diversely, as they assessed higher stress level among families with lower economic status. The result diversity between this study and Lever, Gallo and Goyal’s study research might be because of existing difference of the samples. For example, Goyal’s respondents were pregnant women in third trimester of pregnancy, while all pregnant women in any age of pregnancy entered in the present study. Moreover, Lever et al. included both men and women in their study for assessing the impact of SES on stress rate, however, this study only applied for pregnant women. Furthermore, Gallo et al. entered women in the age of 40-65 years old in their study, whereas the mean of women’s age in the present study was 27±4.8 years. Apart from these same findings, Kingston et al. (2012) realized that there is a significant relationship and direct correlation between monthly family income and perceived stress during pregnancy. Surprisingly, they expressed that pregnant women from low income families experience lower perceived stress level (Kingston et al., 2012). Also, with having a look at Stancil et al. (2000) study, the Kingston’s claiming could be more approved. They showed that with raising the amount of family income, the pregnancy perceived stress immediately rise up.

In term of education, a number of studies found a reversed correlation between perceived stress during pregnancy and pregnant women’s educational level. They asserted that well-educated women are able to manage their life stress critically (Lau & Yin, 2011). In contrary to them, many researchers claimed that women with greater education could conceive stress rate more than low-educated women (Gallo et al., 2012; Goyal et al., 2010; Kingston et al., 2012; Woods, Melville, Guo, Fan, & Gavin, 2010).

Stancil et al. (2000) conducted a survey assessing the impact of both education and income on pregnancy stress. It is remarkable that rich and low-educated pregnant women experience maternal stress more than poor and well-educated ones.

Consistent with our study, there are some researches affirming that there is no significant relationship between SES and stress in domain of education specially (Larsson, Sydsjö, & Josefsson, 2004; Zelkowitz et al., 2004).

Surprisingly, we found a significant relationship between the kind of husbands’ job and perceived stress by pregnant mothers. This result is consistent with Farkas and Valdes’s (2010) finding. They point out that the kind of partner’s job influences stress level in mother. In other words, with raising the class of husband’s job, mother experience the lower rate of stress.

The reason of our inconsistent results with many researchers would be the relatively heterogeneous population participating in the present study. As mentioned before, all pregnant women had entered in this study were from various conditions economically and educationally. Another thing is that our respondents were selected from women in first or second pregnancy and small families as well. As in Iranians’ culture, having child is more important than financial and social situation. Maybe the results from extend families (3 or more children) be different from our study totally.

This study had some strength points: Firstly, according our knowledge, this is the first study examining the relationship of SES and pregnancy stress in Iran. In addition, we tried to select a research area with mixed low and high socioeconomic status to capture much more real outcomes.

## 5. Conclusions

As the number of conducted studies on pregnant women to address their stress and relevant factors are very scarce in Iran, it can be a responsibility of government and relevant organizations to finance further investigations in this understudied field to extent the results to pregnant women in other areas.

## Limitations

The present study had some limitations that should be considered for further studies. The number of questions for specific pregnancy stress questionnaire was too many. Therefore, some respondents got exhausted to reply them completely. Secondly, this study was the cross-sectional study and the mood of women at the time of filling the questionnaire might had influence on their responds. It seems be a good idea to conduct future studies with longitudinal approach to overcome this assumed bias. Finally, with a quantitative research, we cannot immerse in depth of women’s feeling about their stress and their financial and social needs. Thus, we offer further research approaching qualitative method (face to face interview, focus group, open-ended questionnaire and in-depth interview) to tackle this obstacle.
